# Contribution of Afferent Feedback to Adaptive Hindlimb Walking in Cats: A Neuromusculoskeletal Modeling Study

**DOI:** 10.3389/fbioe.2022.825149

**Published:** 2022-04-08

**Authors:** Yongi Kim, Shinya Aoi, Soichiro Fujiki, Simon M. Danner, Sergey N. Markin, Jessica Ausborn, Ilya A. Rybak, Dai Yanagihara, Kei Senda, Kazuo Tsuchiya

**Affiliations:** ^1^ Department of Aeronautics and Astronautics, Graduate School of Engineering, Kyoto University, Kyoto Daigaku-Katsura, Kyoto, Japan; ^2^ Department of Physiology, School of Medicine, Dokkyo Medical University, Tochigi, Japan; ^3^ Department of Neurobiology and Anatomy, Drexel University College of Medicine, Philadelphia, PA, United States; ^4^ Department of Life Sciences, Graduate School of Arts and Sciences, The University of Tokyo, Tokyo, Japan

**Keywords:** walking, cat, neuromusculoskeletal model, central pattern generator, afferent feedback

## Abstract

Mammalian locomotion is generated by central pattern generators (CPGs) in the spinal cord, which produce alternating flexor and extensor activities controlling the locomotor movements of each limb. Afferent feedback signals from the limbs are integrated by the CPGs to provide adaptive control of locomotion. Responses of CPG-generated neural activity to afferent feedback stimulation have been previously studied during fictive locomotion in immobilized cats. Yet, locomotion in awake, behaving animals involves dynamic interactions between central neuronal circuits, afferent feedback, musculoskeletal system, and environment. To study these complex interactions, we developed a model simulating interactions between a half-center CPG and the musculoskeletal system of a cat hindlimb. Then, we analyzed the role of afferent feedback in the locomotor adaptation from a dynamic viewpoint using the methods of dynamical systems theory and nullcline analysis. Our model reproduced limb movements during regular cat walking as well as adaptive changes of these movements when the foot steps into a hole. The model generates important insights into the mechanism for adaptive locomotion resulting from dynamic interactions between the CPG-based neural circuits, the musculoskeletal system, and the environment.

## 1 Introduction

Mammalian locomotion is generated by the central pattern generators (CPGs) located in the spinal cord, which control the movements of each limb (Grillner, 1981; Rossignol, 1996; Orlovsky et al., 1999; Rossignol et al., 2006). CPGs produce the basic locomotor rhythm and create alternating flexor and extensor motoneuron (Mn) activities. Furthermore, they integrate afferent feedback signals to achieve adaptive locomotion.

CPGs can operate without afferent feedback and continuous electrical stimulation of the midbrain locomotor region in immobilized decerebrate cats produces “fictive locomotion” consisting of rhythmic alternating activation of flexor and extensor Mns similar to that occurring during normal locomotion in intact animals (Rossignol, 1996). Such fictive locomotor preparations have been used to investigate the mechanism for the adaptive regulation of locomotor patterns by somatosensory afferent feedback. These studies have shown that stimulation of flexor and extensor sensory afferents can delay or advance flexion-to-extension or extension-to-flexion transitions depending on the timing of the afferent stimulation (Guertin et al., 1995; Perreault et al., 1995; McCrea, 2001; Stecina et al., 2005). In our previous modeling work (Fujiki et al., 2019), we used a half-center type CPG model and analyzed its responses to afferent stimulation using dynamic systems theory based on nullclines. This previous study was limited to the consideration of only neural responses without interaction between the neural and musculoskeletal systems.

However, the mammalian locomotion is a complex phenomenon involving dynamic interactions between the neural circuits, the musculoskeletal system, and the environment. To investigate these interactions and the roles of afferent feedback in adaptive locomotion, investigators studied locomotion in both intact and decerebrate or spinal animals walking on a treadmill by applying various disturbances or introducing holes and obstacles on the treadmill to disturb normal locomotion (Hiebert et al., 1994; Lam and Pearson, 2001; Drew et al., 2002). Although these studies have revealed adaptive responses to such disturbances, the adaptation mechanisms based on the interactions between the neural system, musculoskeletal system, and environment remain poorly understood.

Here, we extended our previous models (Markin et al., 2010, 2016), which integrated a two-level half-center CPG and a musculoskeletal model of the cat hindlimbs and simulated steady walking, to investigate the mechanism of locomotor adaptation in response to perturbations from a dynamical viewpoint. We determined the necessary model parameters through optimization that allows the model to reproduce regular walking movements on a treadmill. We considered locomotion with the presence of holes in the walking surface, which evoked locomotor disturbances that allowed us to analyze the roles of afferent feedback from flexor and extensor muscles to stabilize locomotion. Particularly, the model realistically reproduced adaptive changes in locomotor characteristics observed when the paw of a cat hindlimb stepped into a hole (Hiebert et al., 1994). The model suggests an afferent feedback-based mechanism for locomotor adaptation, which we analyzed based on dynamical systems theory methods using nullclines (Fujiki et al., 2019). Our simulation and analysis provide important insights into the mechanisms for adaptive locomotion based on dynamic interactions between the neural system, the musculoskeletal system, and the environment.

## 2 Model

**FIGURE 1:**
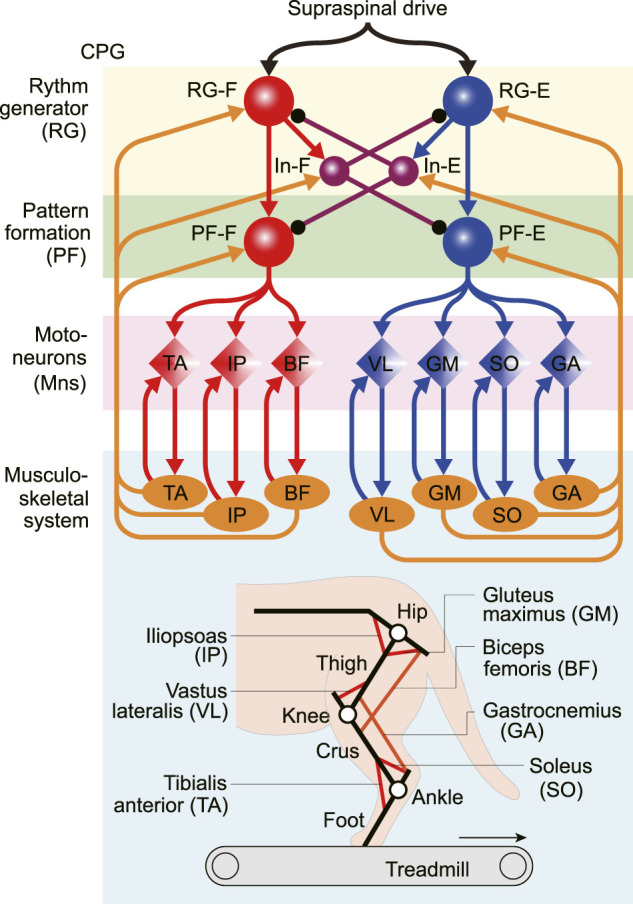
Neuromusculoskeletal model of a cat hindlimb composed of a neural network and a musculoskeletal model. The neural network model consists of a two-level central pattern generator (CPG) with rhythm generating (RG) and pattern formation (PF) circuits and motoneurons (Mns). This network generates motor commands through Mns and receives afferent feedback signals from the musculoskeletal model (orange arrows). The musculoskeletal model, which consists of three rigid links (black lines) and seven muscles (red and orange lines), walks on a treadmill.

Figure 1 shows our neuromusculoskeletal model, which consists of a musculoskeletal model of a cat hindlimb and a spinal CPG model to drive the musculoskeletal model.

### 2.1 Musculoskeletal Model

The skeletal model is two-dimensional and consists of three rigid links representing the thigh, crus, and foot. These links are connected by the knee and ankle joints, and the hip joint is fixed above a treadmill. The model walks on the treadmill with a belt speed of 0.4 m/s based on Prilutsky et al. (2016). When the thigh, crus, and foot are in a straight line and parallel to the vertical line, the hip angle is 135° and the knee and ankle angles are both 180°. The joint angles increase as the joints are extending. The contact between the limb tip and treadmill were modeled using viscoelastic elements. We derived the equations of motion for the skeletal model using Lagrangian equations, where we used the same physical parameters for the skeletal model as those in Ekeberg and Pearson (2005), and solved the equations numerically using the fourth-order Runge-Kutta method with a time step of 0.04 ms.

The skeletal model is driven by seven Hill-type muscles including five uni-articular muscles which are: hip flexor (iliopsoas, IP), hip extensor (gluteus maximus, GM), knee extensor (vastus lateralis, VL), ankle flexor (tibialis anterior, TA), ankle extensor (soleus, SO), and two bi-articular muscles including hip extensor/knee flexor (biceps femoris, BF) and knee flexor/ankle extensor (gastrocnemius, GA). We assumed that the moment arms of all muscles are constant. Each muscle generates the muscle tension through contractile and passive elements. The muscle model consisting of contractile and passive elements is based on the same description and parameters in Ekeberg and Pearson (2005). Specifically, the muscle force $F_{m}\left(m\in \left\{M\right\}=\left\{IP,GM,VL,TA,SO,BF,GA\right\}\right)$ is given by

$$F_{m}=F_{m}^{\text{max}}\left(a_{m}F_{m}^{l}F_{m}^{v}+F_{m}^{p}\right)$$ (1)

where $F_{m}^{\text{max}}$ is the maximum isometric force, *a*_*m*_ is the muscle activation $\left(0\leq a_{m}\leq 1\right)$ , $F_{m}^{l}$ is the force-length relationship, $F_{m}^{v}$ is the force-velocity relationship, and $F_{m}^{p}$ is the passive component. The muscle lengths were normalized by $l_{m}^{\text{max}}$ , which was set so that all uni-articular muscles had a length of 85% of $l_{m}^{\text{max}}$ and all bi-articular muscles were at 75% at a neutral posture with the hip at 65°, the knee at 90°, and the ankle at 100°. In addition, 2° of joint motion corresponded to 1*%* of muscle length change, except for the GA muscle, where 1.5° at the ankle or 4.5° at the knee was required. The muscle contractile velocities were normalized by $l_{m}^{\text{max}}$ as well.

The muscle activation *a*_*m*_ $\left(m\in \left\{M\right\}\right)$ determines the muscle tension generated by the contractile element and the dynamics of *a*_*m*_ is given by a low-pass filter (Yakovenko et al., 2004) as follows:

$${\dot{a}}_{m}+\frac{1}{\tau_{\text{act}}}\left(\frac{\tau_{\text{act}}}{\tau_{\text{dact}}}+\left[1-\frac{\tau_{\text{act}}}{\tau_{\text{dact}}}\right]u_{m}\right)a_{m}=\frac{1}{\tau_{\text{act}}}u_{m}$$ (2)

where *τ*_act_ and *τ*_dact_ are activation and deactivation time constants (20 and 32 ms, respectively), and *u*_*m*_ is the motor command determined from the activities of the corresponding Mn of the CPG model.

### 2.2 CPG Model

The locomotor CPG has been suggested to consist of hierarchical networks, which include rhythm generator (RG) and pattern formation (PF) networks (Rybak et al., 2006a,b). The RG network generates the rhythmic activities while the PF network generates the spatiotemporal patterns of motor commands. For the RG model, we used two neuron populations of flexor and extensor centers (RG-F and RG-E), which receive a supraspinal drive, and two populations of inhibitory interneurons (In-F and In-E), which provide mutual inhibition between the RG-F and RG-E centers. The PF was modeled using two neuron populations of flexor and extensor centers (PF-F and PF-E). The PF-F and PF-E neuron populations receive the excitatory input from the RG-F and RG-E neuron populations, and inhibitory input from the In-E and In-F neuron populations, respectively. Seven Mn populations provide activation for each muscle in the musculoskeletal model (Mn*-m*, $m\in \left\{M\right\}$ ). The Mns of flexor muscles Mn-IP, Mn-TA, and Mn-BF receive excitatory input from the PF-F neuron populations, while those of extensor muscles Mn-GM, Mn-VL, Mn-SO, and Mn-GA receive the excitatory input from the PF-E neuron populations. Synaptic interactions between all neuron populations are shown in Figure 1.

Each population is described as an activity-based (non-spiking) neuron model (Ermentrout, 1994; Markin et al., 2010; Molkov et al., 2015; Danner et al., 2016, 2017). The state of each neuron is characterized by the membrane potential *V*_*i*_ for $i\in \left\{RG\right\}$ , $\left\{In\right\}$ , $\left\{PF\right\}$ , and $\left\{Mn\right\}$ , where {RG} ={RG-F, RG-E}, {In} ={In-F, In-E}, {PF} ={PF-F, PF-E}, and {Mn} $=\left\{Mn‐m\;|\;m\in \left\{M\right\}\right\}$ . The RG, PF, and Mn neurons incorporate a persistent (slowly-inactivating) sodium current that defines the intrinsic rhythmogenic properties of these neurons. The intrinsic oscillations in the RG, PF, and Mn neurons depend on the variable *h*_*i*_ ( $i\in \;\left\{RG\right\}$ , $\left\{PF\right\}$ , $\left\{Mn\right\}$ ) that defines the slow inactivation of the persistent sodium channel. The RG-F and RG-E neurons can produce rhythmic activities. However, if uncoupled, the RG-E neuron is in the tonic regime due to the supraspinal drive and produces sustained activity. Rhythmic oscillations of the RG neurons are defined by the RG-F neuron, which provides rhythmic inhibition of the RG-E neuron through the In-F neuron. The supraspinal drive to the RG-F neuron determines the oscillation frequency. When the PF and Mn neurons are uncoupled, they do not produce rhythmic activities due to the relatively low maximum conductance of the sodium current. Instead, these neurons produce rhythmic activities through the excitatory inputs from the correspondent RG neurons.

For the state variable for this model, we used $V={\left[V_{\left\{RG\right\}},V_{\left\{In\right\}},V_{\left\{PF\right\}},V_{\left\{Mn\right\}}\right]}^{T}$ and $h={\left[h_{\left\{RG\right\}},h_{\left\{PF\right\}},h_{\left\{Mn\right\}}\right]}^{T}$ . The dynamics of the membrane potential *V*_*i*_ is described as

$$C{\dot{V}}_{i}=\left\{\begin{array}{cc} -I_{NaP}\left(V_{i},h_{i}\right)-I_{Leak}\left(V_{i}\right)-I_{SynE}^{i}\left(V\right)-I_{SynI}^{i}\left(V\right)\; & i\in \left\{RG\right\},\left\{PF\right\},\left\{Mn\right\},\\ -I_{Leak}\left(V_{i}\right)-I_{SynE}^{i}\left(V\right)-I_{SynI}^{i}\left(V\right)\; & \;i\in \left\{In\right\}, \end{array}\right.$$ (3)

where *C* is the membrane capacitance, *I*_NaP_ is the persistent sodium current, *I*_Leak_ is the leakage current, and $I_{SynE}^{i}$ and $I_{SynI}^{i}$ are the respective currents in excitatory synapses and inhibitory synapses. The ionic current *I*_NaP_ and leakage current *I*_Leak_ are described as

$$\begin{array}{cc} I_{NaP}\left(V_{i},h_{i}\right) & ={\hat{g}}_{NaP}^{i}m_{\text{NaP}}\left(V_{i}\right)h_{i}\left\{V_{i}-E_{\text{Na}}\right\}\;i\in \left\{RG\right\},\left\{PF\right\},\left\{Mn\right\},\\ I_{Leak}\left(V_{i}\right) & ={\hat{g}}_{\text{Leak}}^{i}\left\{V_{i}-E_{\text{Leak}}^{i}\right\}\;i\in \left\{RG\right\},\left\{In\right\},\left\{PF\right\},\left\{Mn\right\}, \end{array}$$ (4)

where ${\hat{g}}_{NaP}^{i}$ and ${\hat{g}}_{Leak}^{i}$ are the maximum conductances of the corresponding currents, and *E*_Na_ and $E_{Leak}^{i}$ the reversal potentials. In addition, *m*_NaP_ is the activation of the sodium channel of the RG, PF, and Mn neurons and is described as

$$m_{\text{NaP}}\left(V_{i}\right)=\frac{1}{1+\exp\left(-\frac{V_{i}+40.0}{6.0}\right)}\;i\in \left\{RG\right\},\left\{PF\right\},\left\{Mn\right\}.$$ (5)

The dynamics of the inactivation of the sodium channel *h*_*i*_ of the RG, PF, and Mn neurons is given by

$$\tau \left(V_{i}\right){\dot{h}}_{i}=h_{\infty }\left(V_{i}\right)-h_{i}\;i\in \left\{RG\right\},\left\{PF\right\},\left\{Mn\right\},$$ (6)

where

$$h_{\infty }\left(V_{i}\right)=\frac{1}{1+\exp\left(\frac{V_{i}+45.0}{4.0}\right)},$$ (7)

$$\tau \left(V_{i}\right)=320+\frac{320}{\cosh\left(\frac{V_{i}+35.0}{15.0}\right)}\;ms\;i\in \left\{RG\right\},\left\{PF\right\},\left\{Mn\right\}$$

The currents generated by the synapses $I_{SynE}^{i}$ and $I_{SynI}^{i}$ are given by

$$\begin{array}{cc} I_{SynE}^{i} & ={\hat{g}}_{\text{SynE}}\left\{V_{i}-E_{\text{SynE}}\right\}\left\{\underset{j\in \left\{RG\right\},\left\{In\right\},\left\{PF\right\}}{\sum }\alpha_{ij}f\left(V_{j}\right)+\gamma_{i}d+s_{i}\right\},\\ I_{SynI}^{i} & ={\hat{g}}_{\text{SynI}}\left\{V_{i}-E_{\text{SynI}}^{i}\right\}\left\{\underset{j\in \left\{RG\right\},\left\{In\right\},\left\{PF\right\}}{\sum }\beta_{ij}f\left(V_{j}\right)\right\}\;i\in \left\{RG\right\},\left\{In\right\},\left\{PF\right\},\left\{Mn\right\}, \end{array}$$ (8)

where ${\hat{g}}_{SynE}$ and ${\hat{g}}_{SynI}$ are the maximum conductances of the corresponding currents; *E*_SynE_ and $E_{SynI}^{i}$ are the reversal potentials of the corresponding currents; *d* is the tonic drive from the supraspinal region; *s*_*i*_ is the afferent feedback from the musculoskeletal model (as determined in Section 4.1); and *α*_*ij*_, *β*_*ij*_, and *γ*_*i*_ are the weight coefficients, where *β*_*ij*_ = 0 for $i\in \left\{Mn\right\}$ and *γ*_*i*_ = 0 for $i\in \left\{PF\right\}$ and $\left\{Mn\right\}$ . Moreover, the output function *f* translates *V* into the integrated population activity and is given by

$$f\left(V_{i}\right)=\left\{\begin{array}{cc} 0\; & V_{i}<V_{th},\\ \left(V_{i}-V_{th}\right)/\left(V_{max}-V_{th}\right)\; & V_{th}<V_{i}<V_{max},\\ 1\; & V_{max}<V_{i}, \end{array}\right.$$ (9)

where *V*_th_ and *V*_max_ are the lower and upper threshold potentials, respectively. The motor command $u_{m}\;\left(m\in \left\{M\right\}\right)$ is given by $u_{m}=f\left(V_{\text{Mn}‐m}\right)$ . Based on Fujiki et al. (2019) and Markin et al. (2010), we determined the parameters for the CPG model (see Appendix A) except for *α*_*ij*_, *β*_*ij*_, and *γ*_*i*_ for $i\in \left\{Mn\right\}$ , which we determined through optimization, as described in Section 4.2.

### 2.3 Calculation of Nullcline

The nullcline is a set of points at which the derivative of a differential equation is equal to zero. It reflects the structure of the solution of the differential equation. After the CPG model was integrated with the musculoskeletal model to achieve steady walking, we used a nullcline-based method, as in our previous work (Fujiki et al., 2019), to investigate the mechanism of the response of the CPG model to a disturbance during steady walking. Specifically, the state of the CPG model is given by $\left(V,h\right)$ , and the nullclines for the RG neurons are given by

$$\left\{\begin{array}{c} N_{i}^{V}=\left\{\left(V,h\right)|\;{\dot{V}}_{i}=0\right\},\;\\ N_{i}^{h}=\left\{\left(V,h\right)|\;{\dot{h}}_{i}=0\right\},\; \end{array}\right.\;i\in \left\{RG\right\}.$$ (10)

To clarify the dynamics of each RG neuron, we focused on the *V*_*i*_ *-h*_*i*_ space $\left(i\in \left\{RG\right\}\right)$ for the nullclines by assuming that the other variables $V_{j}\left(j\in \left\{RG\right\},\left\{In\right\},\left\{PF\right\},\left\{Mn\right\},j\neq i\right)$ and $h_{k}\left(k\in \left\{RG\right\},\left\{PF\right\},\left\{Mn\right\},k\neq i\right)$ are stably oscillating during steady walking. Therefore, we modify $N_{i}^{V}$ and $N_{i}^{h}$ in Eq. 10 as

$$\begin{array}{l} \begin{array}{c} \left\{\begin{array}{c} {\hat{N}}_{i}^{V}=\left\{\left(V_{i},h_{i}\right)|\;{\dot{V}}_{i}=0,V_{j}=V_{j}^{*},h_{k}=h_{k}^{*}\right\},\;\\ {\hat{N}}_{i}^{h}=\left\{\left(V_{i},h_{i}\right)|\;{\dot{h}}_{i}=0,V_{j}=V_{j}^{*},h_{k}=h_{k}^{*}\right\},\; \end{array}\right.\\ i\in \left\{RG\right\},\;j\in \left\{RG\right\},\left\{In\right\},\left\{PF\right\},\left\{Mn\right\},\;j\neq i,\;k\in \left\{RG\right\},\left\{PF\right\},\left\{Mn\right\},\;k\neq i, \end{array} \end{array}$$ (11)

where *x* ^∗^ indicates *x* for the stable oscillation during steady walking.

**FIGURE 2:**
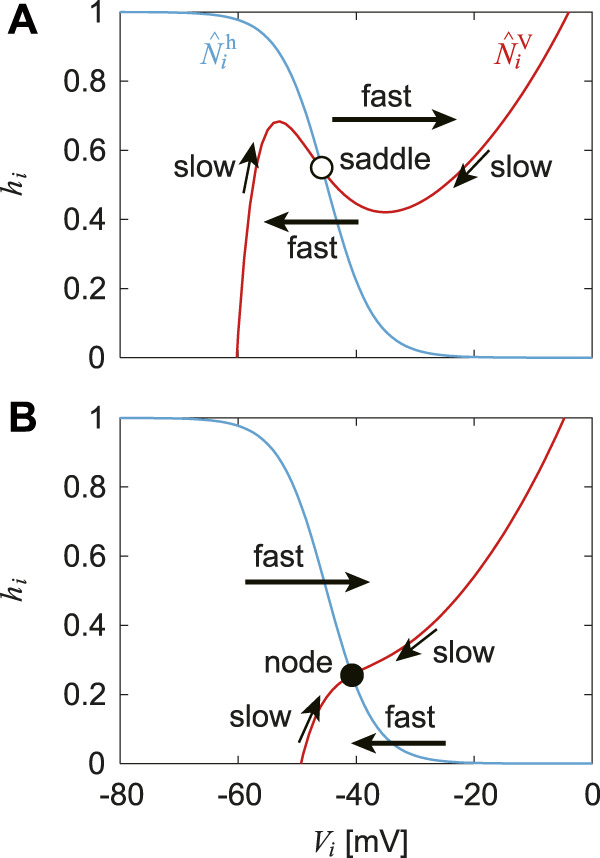
Nullclines ${\hat{N}}_{i}^{V}$ and ${\hat{N}}_{i}^{h}$ for $i\in \left\{RG\right\}$ of RG neurons in the phase plane and fast and slow dynamics that the state (*V*_*i*_, *h*_*i*_) for $i\in \left\{RG\right\}$ follows. **(A)** ${\hat{N}}_{i}^{V}$ has two different inflection points and intersection with ${\hat{N}}_{i}^{h}$ is saddle equilibrium point. **(B)** ${\hat{N}}_{i}^{V}$ changes monotonically and intersection with ${\hat{N}}_{i}^{h}$ is stable node equilibrium point. Bold and thin arrows represent fast and slow dynamics, respectively.

While ${\hat{N}}_{i}^{h}\;\left(i\in \left\{RG\right\}\right)$ has a sigmoid shape and does not change with time, ${\hat{N}}_{i}^{V}\;\left(i\in \left\{RG\right\}\right)$ has a cubic curve shape and changes by afferent feedback and input from other neurons. Specifically, ${\hat{N}}_{i}^{V}$ mainly has the two different situations shown in Figures 2A,B, one with two different inflection points at which the sign of the slope changes, and one with a monotonic variation. In the former case (Figure 2A), the intersection of ${\hat{N}}_{i}^{V}$ and ${\hat{N}}_{i}^{h}$ is a saddle equilibrium point. Because the time constant for the dynamics of $V_{i}\;\left(i\in \left\{RG\right\}\right)$ is smaller than that for $h_{i}\;\left(i\in \left\{RG\right\}\right)$ , the following two features are present (Fujiki et al., 2019; Molkov et al., 2015; Spardy et al., 2011): 1. near where ${\hat{N}}_{i}^{V}$ has positive slope, the state (*V*_*i*_, *h*_*i*_) is slowly attracted to the inflection point along ${\hat{N}}_{i}^{V}$ (slow dynamics) and 2. near the inflection points, the state (*V*_*i*_, *h*_*i*_) jumps to the opposite part of ${\hat{N}}_{i}^{V}$ with a positive slope (fast dynamics). In the latter case (Figure 2B), the intersection of ${\hat{N}}_{i}^{V}$ and ${\hat{N}}_{i}^{h}$ is a stable node equilibrium point having the following two features (Spardy et al., 2011; Fujiki et al., 2019): 1. when the state (*V*_*i*_, *h*_*i*_) is away from ${\hat{N}}_{i}^{V}$ , it is quickly attracted to ${\hat{N}}_{i}^{V}$ (fast dynamics), and 2. near ${\hat{N}}_{i}^{V}$ , the state (*V*_*i*_, *h*_*i*_) is slowly attracted to the stable node along ${\hat{N}}_{i}^{V}$ (slow dynamics). As described above, the switch between fast and slow dynamics depends on the relationship between the state and the nullcline, and adaptive responses are achieved through the changes in ${\hat{N}}_{i}^{V}$ by afferent feedback.

## 3 Verifying the CPG Model by Phase-Dependent Response in Fictive Locomotion

**FIGURE 3:**
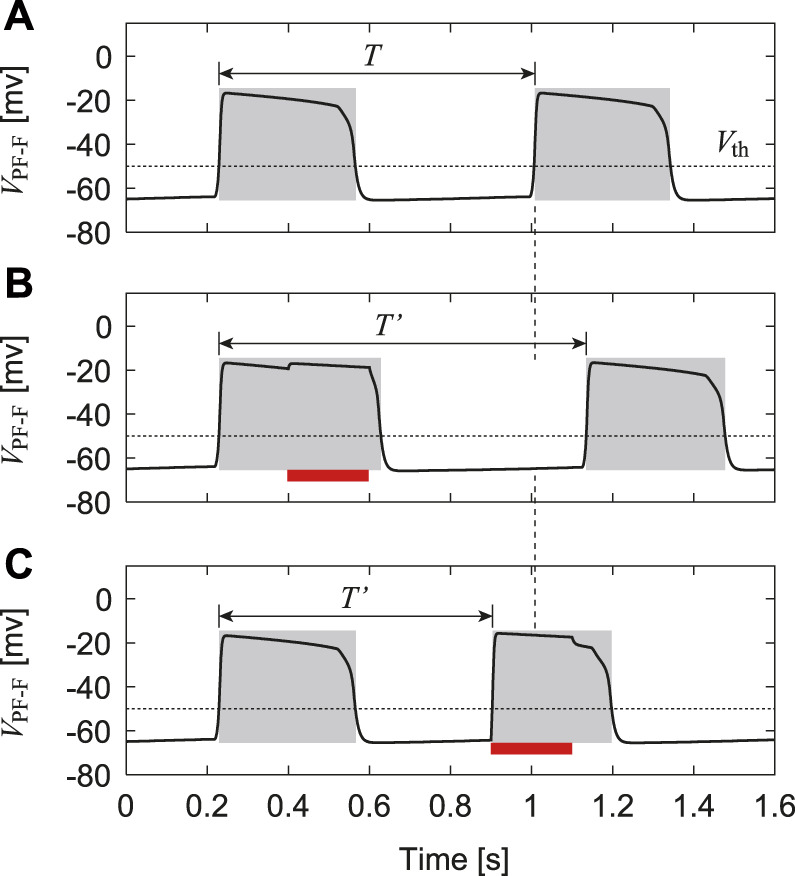
Change in membrane potential of PF-F neuron by stimulation: **(A)** No stimulation. **(B)** Stimulation to the flexor side during the active phase, which increases the duration of the current active phase and cycle (*T*′ > *T*). **(C)** Stimulation to the flexor side during the silent phase, which initiates the active phase and decreases the cycle duration (*T*′ < *T*). Red bars indicate the application of stimulation. Gray regions indicate active phases.

Even when our CPG model is separated from the musculoskeletal model (or receives no feedback signals), it produces rhythmic activities and exhibits stable oscillations, as shown in Figure 3. Based on these oscillations, we define the active phase for each neuron as the time interval during which the neuron’s potential is higher than *V*_th_, and the silent phase as the time interval when the potential is lower than *V*_th_. We also define the cycle period *T* as the time interval between two consecutive onsets of the active phase of the PF-F neuron and the phase of the oscillation as *ϕ* = 2*πt*/*T* ∈ [0, 2*π*).

To verify our CPG model, we investigated the phase-dependent response of the CPG activities based on previous studies (Demir et al., 1997; Fujiki et al., 2019) and compared the results with those obtained by experiments with fictive locomotion in cats (Duysens, 1977; Schomburg et al., 1998). Specifically, we used only the CPG model (RG, In, and PF neurons) separated from the musculoskeletal model. After the oscillation of the CPG model stabilized, we applied a 200-ms stimulus to the flexor (RG-F, In-F, and PF-F) or extensor (RG-E, In-E, and PF-E) neurons, where the intensity of the stimulation was set as follows: *s*_RG-F_ = *s*_In-F_ = *s*_PF-F_ = 0.2 and *s*_RG-E_ = *s*_In-E_ = *s*_PF-E_ = 0 for the stimulation to the flexor side and *s*_RG-F_ = *s*_In-F_ = *s*_PF-F_ = 0.0 and *s*_RG-E_ = *s*_In-E_ = *s*_PF-E_ = 0.2 for the stimulation to the extensor side in Eq. 8. Suppose that the neuron activity is perturbed by stimulation with a phase *ϕ*_s_ ∈ [0, 2*π*) and the period of the PF-F neuron changes from *T* to *T*′(*ϕ*_s_), as shown in Figure 3. To explain the phase shift of the neuron activity in response to the stimulation, we define

$$\Delta \left(\phi_{s}\right)=2\pi \frac{T^{\prime }\left(\phi_{s}\right)-T}{T}$$ (12)

**FIGURE 4:**
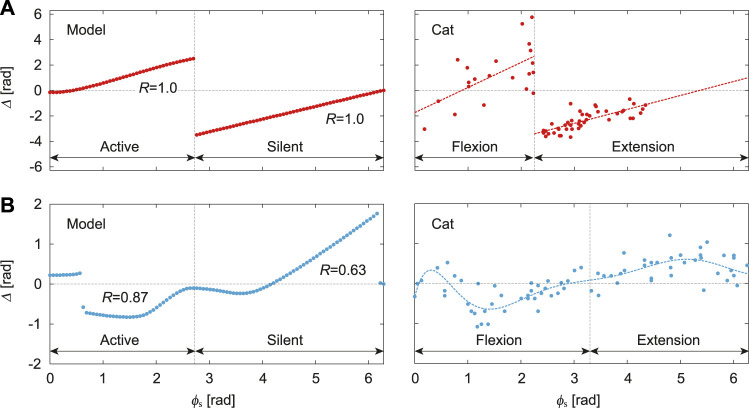
Phase-dependent response of CPG model to a stimulation. **(A)** Response of PF-F neuron in our model to stimulation of the flexor side compared with the response against flexor muscle stimulation during fictive locomotion in spinal cats [adapted from Schomburg et al. (1998)], where the dotted lines are approximate functions using a first order polynomial for each flexion and extension phase. **(B)** Response of PF-F neuron of our model to stimulation of the extensor side compared with the response against extensor muscle stimulation during fictive locomotion in decerebrate cats [adapted from Duysens (1977)], where the dotted line is an approximate function using an eighth order polynomial. *R* is the correlation coefficient between the active/silent phase of the simulation results and flexion/extension phase of the approximate function.

Figure 4A shows the phase shift Δ of the PF-F neuron activity after the stimulation of sensory inputs on the flexor side at *ϕ*_s_. When the stimulation was applied during the silent phase of the PF-F neuron (2.70 ≤ *ϕ*_s_ ≤ 2*π*), it caused an earlier transition to the active phase, and this advanced start decreased with *ϕ*_s_. In contrast, almost no phase shift occurred when the stimulation was applied at the beginning of the active phase of the PF-F neuron (0 ≤ *ϕ*_s_ ≤ 0.44). However, the neuron activity was delayed by the stimulation during the middle and end of the active phase (0.44 ≤ *ϕ*_s_ ≤ 2.70). These trends were similar to those observed during fictive locomotion in spinal cats (Schomburg et al., 1998; Frigon et al., 2010), as shown in Figure 4A.

Figure 4B shows Δ after stimulation of the extensor side. The active and silent phase of the PF-F neuron corresponds to the silent and active phase, respectively, of the PF-E neuron. The neuron activity was advanced at the middle of the silent phase of the PF-E neuron (0.63 ≤ *ϕ*_s_ ≤ 2.64) and was delayed at the end of the active phase of the PF-E neuron (4.27 ≤ *ϕ*_s_ ≤ 6.16). The response of the stimulation of the extensor side was qualitatively similar to that for the flexor side. Moreover, the trends were consistent with those observed during fictive locomotion in decerebrate cats (Duysens, 1977; Schomburg et al., 1998; Frigon et al., 2010), as shown in Figure 4B. These results verify the validity of our CPG model.

## 4 Determining the Motor Control Model by Optimization to Reproduce Normal Walking

We integrated the CPG and musculoskeletal models to determine the remaining parameters for the CPG model through an optimization to produce normal walking on the treadmill.

### 4.1 Afferent Feedback From the Musculoskeletal Model

We determined the afferent feedback *s*_*i*_ in Eq. 8 based on Markin et al. (2010) as follows:

$$s_{i}=\underset{m\in \left\{M\right\}}{\sum }\left(k_{im}^{v}{\left(v_{m}^{\mathrm{norm}}\right)}^{0.6}+k_{im}^{d}d_{m}^{\mathrm{norm}}+k_{im}^{f}F_{m}^{\mathrm{norm}}\right)\;i\in \left\{RG\right\},\left\{In\right\},\left\{PF\right\},$$ (13)

where

$$v_{m}^{\text{norm}}=\left\{\begin{array}{cc} v_{m}/l_{m}^{\text{max}}\; & v_{m}>0,\\ 0\; & otherwise; \end{array}\right.$$ (14)

$$d_{m}^{\text{norm}}=\left\{\begin{array}{cc} \left(l_{m}-l_{\text{th}}\right)/l_{m}^{\text{max}}\; & l_{m}>l_{\text{th}},\\ 0\; & otherwise; \end{array}\right.$$ (15)

$$F_{m}^{\text{norm}}=\left\{\begin{array}{cc} F_{m}/F_{m}^{\text{max}}\; & F_{m}>0,\\ 0\; & otherwise; \end{array}\right.$$ (16)

$$l_{\text{th}}=0.9l_{m}^{\text{max}};$$ (17)

*v*_*m*_, *l*_*m*_, and *F*_*m*_ are the velocity, length, and force of muscle *m*, respectively, and $k_{im}^{v}$ , $k_{im}^{d}$ , and $k_{im}^{f}$ are the corresponding weight coefficients. The three terms on the right-hand side of Eq. 13 represent the velocity, length, and force feedback from muscle *m*. The coefficients $k_{im}^{v}$ , $k_{im}^{d}$ , and $k_{im}^{f}$ are given as follows: for the flexor side (*i* = RG-F, In-F, PF-F),

$$\left(k_{im}^{v},k_{im}^{d},k_{im}^{f}\right)=\left\{\begin{array}{cc} \left(k_{F}^{v},k_{F}^{d},k_{F}^{f}\right)\; & m=IP,TA,BF,\\ \left(0,0,0\right)\; & otherwise; \end{array}\right.$$ (18)

for the extensor side (*i* = RG-E, In-E, PF-E),

$$\left(k_{im}^{v},k_{im}^{d},k_{im}^{f}\right)=\left\{\begin{array}{cc} \left(k_{E}^{v},k_{E}^{d},k_{E}^{f}\right)\; & m=GM,VL,SO,GA,\\ \left(0,0,0\right)\; & otherwise; \end{array}\right.$$ (19)

and for the Mns $\left(i\in \left\{Mn\right\}\right)$ ,

$$\left(k_{im}^{v},k_{im}^{d},k_{im}^{f}\right)=\left\{\begin{array}{cc} \left(\eta k_{F}^{v},\eta k_{F}^{d},\eta k_{F}^{f}\right)\; & i=Mn‐m\left(m=IP,TA,BF\right),\\ \left(\eta k_{E}^{v},\eta k_{E}^{d},\eta k_{E}^{f}\right)\; & i=Mn‐m\left(m=GM,VL,SO,GA\right),\\ \left(0,0,0\right)\; & otherwise. \end{array}\right.$$ (20)

We determined $k_{F}^{v},k_{F}^{d},k_{F}^{f},k_{E}^{v},k_{E}^{d},k_{E}^{f}$ , and *η* through the optimization technique described in the next section.

### 4.2 Optimization to Determine Motor Control Parameters

We integrated the CPG and musculoskeletal models and determined the motor control parameters through an optimization to produce normal walking on the treadmill based on the measured kinematic data for cats (Prilutsky et al., 2016). Specifically, we determined the following 14 parameters; *α*_Mn-IP,PF-F_, *α*_Mn-GM,PF-E_, *α*_Mn-VL,PF-E_, *α*_Mn-TA,PF-F_, *α*_Mn-SO,PF-E_, *α*_Mn-BF,PF-F_, and *α*_Mn-GA,PF-E_ in Eq. 8 (the other *a*_*ij*_’s for $i\in \left\{Mn\right\}$ are 0) and $k_{F}^{v},k_{F}^{d},k_{F}^{f},k_{E}^{v},k_{E}^{d},k_{E}^{f}$ , and *η* in Eqs. 18–20. We used the covariance matrix adaptation evolution strategy (CMA-ES) (Hansen et al., 2003) as the optimization method to minimize the following loss function:

$$\varepsilon =\int_{t_{1}}^{t_{2}}\underset{i\in \left\{J\right\}}{\sum }{\left(\theta_{i}-{\hat{\theta }}_{i}\right)}^{2}dt$$ (21)

where $\left\{J\right\}=\left\{Hip,Knee,Ankle\right\}$ , *θ*_*i*_ and ${\hat{\theta }}_{i}$ are the simulated and measured joint angles, and *t*_1_ and *t*_2_ were determined to evaluate the error for two gait cycles after steady walking was achieved.

**FIGURE 5:**
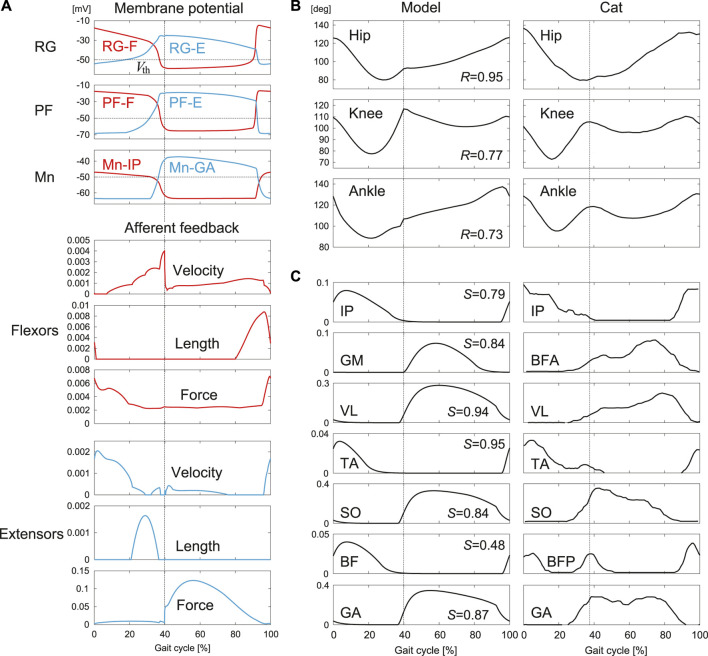
Simulation results for normal walking obtained by the optimization: **(A)** Membrane potentials of CPG neurons and afferent feedback from flexor and extensor muscles. **(B)** Joint angles and **(C)** muscle activations compared with measured data in cat [adapted from Prilutsky et al. (2016)]. *R* is the correlation coefficient and *S* is the cosine similarity. Liftoffs are represented by 0 and 100% in the gait cycle. Vertical lines indicate touchdowns.

Figure 5 shows the results obtained by the optimization. The model parameters determined by the optimization are shown in Appendix B. Figure 5A shows the membrane potentials of the RG, PF, and Mn neurons and the velocity, length, and force feedback of the flexor and extensor muscles. The rhythmic activities of the RG-F and RG-E neurons were transmitted to the Mn neurons through the PF neurons and the afferent feedback was used in normal walking. Figures 5B,C show the joint angles and muscle activations, respectively, compared with the data measured for cats (Prilutsky et al., 2016). The simulated joint angles and muscle activities have patterns similar to those for the measured data. These results verify that our model reproduced regular cat walking on a treadmill through the interactions between the neural system, the musculoskeletal system, and the environment by the optimization.

## 5 Adaptive Response to Stepping Into a Hole During Treadmill Walking

To investigate the role of afferent feedback in adaptive locomotion, we applied a disturbance to our model and compared the responses with those measured for cats. Moreover, we clarified the adaptation mechanism based on dynamic systems theory methods.

### 5.1 Comparison of Simulation Results and Measured Data

Afferent feedback plays important roles in adaptive walking. To clarify these roles, a treadmill with a hole has been used in experiments with cats (Gorassini et al., 1994; Hiebert et al., 1994), where the cat body and forelimbs are supported and the cat walks on the treadmill only with the hindlimbs. When the foot of a hindlimb steps into the hole, it loses ground contact, and an appropriate response based on afferent feedback is required to continue walking. The investigation of the response to stepping into a hole highlights the roles of afferent feedback from the flexor and extensor muscles in generating adaptive walking. Hiebert et al. (1994) showed that when the foot of a hindlimb of a spinal cat entered a hole, the activities of the extensor muscles were shortened and the next onsets of the activities of the flexor and extensor muscles were advanced, which quickly lifted the foot out of the hole. We conducted a simulation of our model in the same situation as Hiebert et al. (1994) using the model parameters determined in the previous section to generate normal walking and compared the results with the measured data from animal experiments. In the simulation, after our model achieved steady walking on a treadmill, we emulated a hole by setting the ground reaction force to zero when the foot touched the treadmill belt until the foot went below the belt and returned back above it.

**FIGURE 6:**
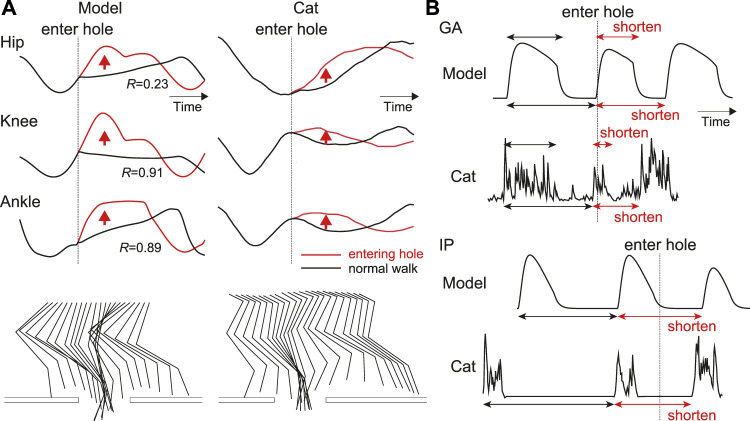
Simulation results when on paw enters a hole compared with those during normal walking: **(A)** Joint angles and stick diagrams compared with measured data [adapted from Hiebert et al. (1994) for entering a hole and from Prilutsky et al. (2016) for normal walking]. These data are shown from the liftoff before entering a hole to the touchdown after entering the hole. *R* is the correlation coefficient of the results after entering the hole. The stick diagrams are shifted to the left by according to the distance traveled by the treadmill belt. **(B)** Activities of IP and GA muscles compared with measured data in spinal cat [adapted from Hiebert et al. (1994)]. Upper arrows indicate the activity duration, and lower ones indicate the intervals between the onsets of current and subsequent activities.

Figure 6 compares the simulation results when the foot entered a hole with those during normal walking. Specifically, Figure 6A shows the joint angles and stick diagrams. When the foot stepped into the hole, the hip, knee, and ankle joints were first extended. After that, these joints were flexed to lift the foot above the treadmill belt and then the foot landed on the belt. This behavior was consistent with that observed in the animal experiment. Figure 6B shows the activation of the IP and GA muscles representing the flexor and extensor muscles, respectively. The activation of the GA muscle was shortened and the onset of the next activation was advanced compared with those in normal walking. The onset of the next activation of the IP muscle was also advanced. These responses were consistent with those in the animal experiment. These results verify that our model reproduced adaptive responses in cats when a foot steps into a hole during walking, even without changing the model parameters, which were determined to reproduce normal walking.

### 5.2 Investigating the Adaptation Mechanism

**FIGURE 7:**
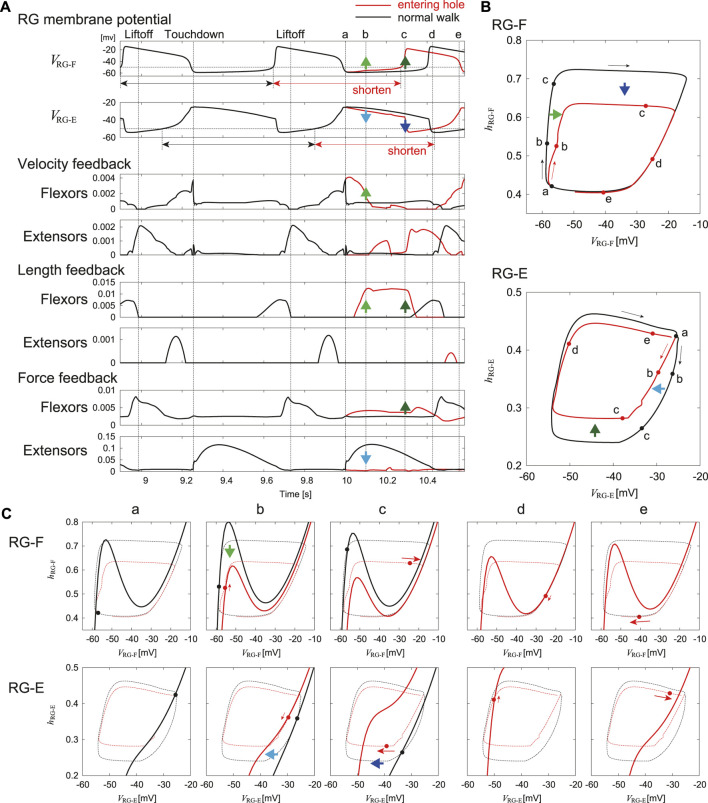
Comparison of the response of RG neurons to a foot entering a hole with steady oscillation during normal walking. **(A)** Time profiles of membrane potentials and afferent feedback from flexor and extensor muscles. “a” indicates the time at which the foot enters the hole (touchdown in normal walking). “b”–“e” indicate the lapsed times of 0.1, 0.29, 0.43, and 0.56 s, respectively, after “a”. **(B)** Trajectories of $\left(V_{RG‐F},h_{RG‐F}\right)$ and $\left(V_{RG‐E},h_{RG‐E}\right)$ in phase planes. **(C)** Changes of nullclines ${\hat{N}}_{RG‐F}^{V}$ and ${\overset{‐}{N}}_{RG‐E}^{V}$ .

We investigated the adaptation mechanism *via* afferent feedback when the foot stepped into a hole based on the nullclines of the RG neurons in the phase plane. Figure 7 compares the stimulation results for the RG neurons between entering a hole and normal walking. Specifically, Figure 7A shows the time profiles of the membrane potentials of the RG-F and RG-E neurons and the velocity, length, and force feedback from the flexor and extensor muscles. Figures 7B,C show the trajectories of the state $\left(V_{RG‐F},h_{RG‐F}\right)$ of the RG-F neuron and the state $\left(V_{RG‐E},h_{RG‐E}\right)$ of the RG-E neuron, and the changes in the nullclines ${\hat{N}}_{RG‐F}^{V}$ of the RG-F neuron and ${\hat{N}}_{RG‐E}^{V}$ of the RG-E neuron (because ${\hat{N}}_{RG‐F}^{h}$ and ${\hat{N}}_{RG‐E}^{h}$ do not change, they are not shown), respectively, in their phase planes. Although a stable limit cycle appeared during normal walking, the trajectories diverged after the foot entered the hole. Therefore, just before the foot entered the hole, which corresponds to the touchdown in normal walking (“a” in Figure 7), the trajectories and nullclines were identical between entering a hole and normal walking.

In the RG-F neuron, the local maximum of the nullcline decreased 0.1 s after entering the hole (“b” in Figure 7) due to the increased velocity and length feedback from the flexor muscles. The trajectory was attracted to the nullcline while following the slow dynamics. As a result, *V*_RG-F_ increased slightly. In the RG-E neuron, the nullcline remained almost unchanged from that in “a” due to the loss of the force feedback from the extensor muscles. In other words, the nullcline moved to the left compared to that in normal walking. The trajectory was attracted to the nullcline while following the slow dynamics. As a result, *V*_RG-E_ decreased slightly.

In the RG-F neuron, the local maximum of the nullcline further decreased 0.29 s after the foot entered the hole (“c” in Figure 7) due to the increase in the length and force feedback from the flexor muscles. The state entered the fast dynamics by passing over the local maximum. As a result, *V*_RG-F_ rapidly underwent a large increase to initiate the next activity. In the RG-E neuron, the nullcline moved significantly to the left due to the increased inhibition from the RG-F neuron, which made the state enter the fast dynamics. As a result, *V*_RG-E_ decreased strongly and the activity was rapidly terminated.

As a result of these changes, the onset of the next activity of the RG-F neuron was advanced and the current activity of the RG-E neuron was shortened. The state $\left(V_{RG‐F},h_{RG‐F}\right)$ of the RG-F neuron and the state $\left(V_{RG‐E},h_{RG‐E}\right)$ of the RG-E neuron followed the slow dynamics in “d” in Figure 7 and the trajectories and nullclines in “e” in Figure 7 almost returned to those of “a” in Figure 7 through the fast dynamics to continue normal walking. These results clarify the mechanims how our model produced adaptive responses during stepping into a hole through the interactions between the neural system, the musculoskeletal system, and the environment using dynamic systems theory based on nullclines.

## 6 Discussion

### 6.1 Adaptive Responses to Entering a Hole

To continue walking after one of its feet steps into a hole, the cat must quickly pull the foot out of the hole and step onto the treadmill belt again. To provide this behavior the CPG should modify the motor commands based on afferent feedback. Similar to normal walking, the extensor muscles start their activity before touchdown to support the body weight. However, when a foot enters a hole without touchdown, the muscle activity excessively extends the hip, knee, and ankle joints. Therefore, for stable walking it is necessary to quickly terminate extension and start flexion. It has been suggested that unloading of ankle extensor muscles and activation of the Ia and II sensory afferents from flexor muscles play an important role in the transition from stance to swing during normal walking in cats (Duysens and Pearson, 1980; Pearson et al., 1992; Hiebert et al., 1996). Studies of locomotion in spinal cats when holes were present showed that the unloading of the extensor muscle due to the lack of touchdown caused early termination of the extensor activity (Hiebert et al., 1994). In our model, this phase transition was provided by the velocity and length dependent (Ia, II) as well as the force dependent (Ib) feedback. Our simulations have shown that the early termination of extensor activity and early initiation of flexor activity can result from unloading of the extensor muscles (“b” in Figure 7C) and increasing the activity of Ia and II afferent feedback from the flexor muscles of knee and ankle (“c” in Figure 7C) when the foot entering into a hole, which is consistent with the animal experiments. It is important to mention that, although the parameters for afferent feedback were determined during optimization performed to reproduce the normal walking, the afferent feedback incorporated in the model allowed the model to reproduce adaptive changes of locomotor characteristics during stepping into a hole. Furthermore, the mechanism responsible for this adaptive response was explained by the transition from the slow to fast dynamics in the RG neurons induced by the changes in the nullclines due to afferent feedback (Figure 7).

The adaptive locomotor behavior during stepping into a hole can be also interpreted based on the phase-dependent response of the CPG activity. Stimulation of extensor afferents during the active phase in fictive locomotion of decerebrate cats was shown to increase and prolong the extensor activity (Duysens, 1977), and our isolated CPG model was able to reproduce the phase-dependent response (Figure 4B). In the model, during in normal walking, Ib feedback from extensors increased during the stance phase due to the ground reaction force, which increased and prolonged the activities of the extensor half center of the CPG (Figure 5B). However, when the foot entered the hole, the force feedback from the extensor muscles was reduced due to the lack of the ground reaction force and the activity of the extensor half center was reduced (Figures 6B, 7A). Similarly, stimulation of flexors afferents during the silent phase in fictive locomotion in decerebrate cats inhibited the extensor activity and initiated transition to flexion (Schomburg et al., 1998), and our isolated CPG model also reproduced these phase-dependent responses (Figure 4A). The flexor muscles in our model were stretched when the foot entered into a hole and afferent feedback from the flexor side increased (Figure 6B), which inhibited the activities of extensor muscles and shortened the stance phase leading to an advanced onset of the swing phase. As a result, the extension of the joints stopped and they were strongly flexed to pull the foot out of the hole.

### 6.2 Limitations and Future Works

In this study, we focused only on simulation of walking with a single hole as a source of locomotor disturbance. This allowed us to investigate the roles of afferent feedback from both flexor and extensor muscles because stepping into a hole caused unloading of extensor muscles and stretching of flexor muscles. We have not considered other walking disturbances tested experimentally, such as the presence of obstacles, cutaneous stimulation, and mechanical blockage of limb flexion (Forssberg et al., 1977; Lam and Pearson, 2001; Drew et al., 2002). In particular, while we focused on a hole without a bottom, a foot can have touchdown in a hole with a bottom depending on the depth, which changes the afferent feedback and phase transition. In the future, we would like to simulate walking with these other disturbances to provide mechanistic understanding of adaptation observed during locomotion. In addition, our model would be useful to investigate the responses when afferent feedback does not work well due to the paralysis and provides wrong information.

Because our present model used only one pair of the PF neurons (a PF-F neuron for the flexor side and a PF-E neuron for the extensor side), the motor commands activated the flexor and extensor muscles in strict alternation. However, in the biological system, some muscles show activity in both swing and stance phases and some muscles are active during only part of one phase (Prilutsky et al., 2016). In addition, muscle synergy analysis suggested that muscle activities during animal walking can be explained by the combination of a few basic patterns (Dominici et al., 2011). Several previous CPG models have been proposed to produce such multiple patterns by increasing the complexity of the PF network (Markin et al., 2016). It has been also suggested that some afferent feedback is projected to the contralateral side of the CPG (McCrea, 2001). We will include these components in a more elaborated model in the future.

Also in this work, we only modeled one hindlimb, which did not allow us to consider interlimb coordination. The latter is also known to play an important role in adaptive walking (Forssberg et al., 1980). Particularly, when a foot of one limb enters a hole, the activity of the extensor muscle of the contralateral limb has been shown to increase to compensate for the weight support (Hiebert et al., 1994). In addition, animals change their gait patterns, such as walking, trotting, and galloping, depending on locomotor speed by controlling the interlimb coordination. A neural model simulating coordination between RG circuits controlling each limb has been proposed (Danner et al., 2016, 2017). These interactions will be included in the future extended model simulating locomotion with two forelimbs and two hindlimbs.

## Data Availability

The raw data supporting the conclusion of this article will be made available by the authors, without undue reservation.

## Appendix

**A Parameters for CPG Model**

Based on Molkov et al. (2015), Danner et al. (2016), Danner et al. (2017), Markin et al. (2010), and Fujiki et al. (2019), we determined the parameters for the synaptic connections *α*_*ij*_, *β*_*ij*_, and *γ*_*i*_ $\left(i\in \left\{RG\right\},\left\{In\right\},\left\{PF\right\},j\in \left\{RG\right\},\left\{In\right\}\right)$ and those for the currents ${\hat{g}}_{Leak}^{i}$ , ${\hat{g}}_{NaP}^{i}$ , $E_{\text{SynI}}^{i}$ , and $E_{\text{Leak}}^{i}$ $\left(i\in \left\{RG\right\},\left\{In\right\},\left\{PF\right\},\left\{Mn\right\}\right)$ as shown in Tables 1, 2, respectively. We used the following parameter values: ${\hat{g}}_{SynE}=10\;nS$ , ${\hat{g}}_{SynI}=10\;nS$ , *E*_SynE_ = − 10 mV, *E*_Na_ = 55 mV, *C* = 20 pF, *V*_th_ = − 50 mV, *V*_max_ = 0 mV, and *d* = 1.0.

**TABLE 1:** Parameters for synaptic connections.

Source *j*	Target neuron *i*					
	RG-F	RG-E	In-F	In-E	PF-F	PF-E
Excitatory connections *α*_*ij*_						
RG-F	0	0	0.4	0	0.7	0
RG-E	0	0	0	0.4	0	0.7
Inhibitory connections *β*_*ij*_						
In-F	0	0.7	0	0	0	2.1
In-E	0.1	0	0	0	0.3	0
Connections from supraspinal drive *γ*_*i*_	0.02	0.15	0	0	0	0

**TABLE 2:** Parameters for currents.

	Target neuron *i*			
	$i\in \left\{RG\right\}$	$i\in \left\{In\right\}$	$i\in \left\{PF\right\}$	$i\in \left\{Mn\right\}$
${\hat{g}}_{Leak}^{i}$	4.5	2.8	1.6	1.6
${\hat{g}}_{NaP}^{i}$	4.5	—	0.5	0.3
$E_{\text{SynI}}^{i}$	−75	−75	−70	−70
$E_{\text{Leak}}^{i}$	−62.5	−60	−64	−64

**B Parameters Determined by Optimization to Generate Normal Walking**

Through the optimization needed to generate normal walking, the parameters for the synaptic connections *α*_*ij*_ $\left(i\in \left\{Mn\right\},j\in \left\{PF\right\}\right)$ were determined as shown in Table 3 and the weight coefficients for afferent feedback were determined as follows: $k_{F}^{v}=0.001$ , $k_{F}^{d}=0.15$ , $k_{F}^{f}=0.077$ , $k_{E}^{v}=0.000\;39$ , $k_{E}^{d}=0.058$ , $k_{E}^{f}=0.2$ , and *η* = 0.27.

**TABLE 3:** Optimized parameters for synaptic connections.

Source *j*	Target motoneuron *i*						
	Mn-IP	Mn-GM	Mn-VL	Mn-TA	Mn-SO	Mn-BF	Mn-GA
Excitatory connections *α*_*ij*_							
PF-F	0.084	0	0	0.074	0	0.077	0
PF-E	0	0.82	0.13	0	0.16	0	0.18
